# The spectrum of rheumatic in-patient diagnoses at a pediatric hospital in Kenya

**DOI:** 10.1186/s12969-016-0131-3

**Published:** 2017-01-14

**Authors:** Angela Migowa, Inés Colmegna, Carol Hitchon, Eugene Were, Evelyn Ng’ang’a, Thomas Ngwiri, John Wachira, Sasha Bernatsky, Rosie Scuccimarri

**Affiliations:** 1 Division of Pediatric Rheumatology, Department of Pediatrics, McGill University Health Centre, 1001 Decarie Boulevard, Room A04.6306, Montreal, QC Canada H4A 3J1; 2 Division of Rheumatology, Department of Medicine, McGill University Health Centre, Montreal, QC Canada; 3 Division of Rheumatology, Department of Internal Medicine, University of Manitoba, Winnipeg, MB Canada; 4 Department of Pediatrics, Gertrude’s Children’s Hospital, Nairobi, Kenya; 5 Department of Pediatrics, The Nairobi Hospital, Nairobi, Kenya; 6 Division of Rheumatology & Division of Clinical Epidemiology, Department of Medicine & Department of Epidemiology, Biostatistics and Occupational Health, McGill University Health Centre, McGill University, Montreal, QC Canada

**Keywords:** Pediatric rheumatology, Global health, Kenya, Juvenile arthritis, Lupus, Kawasaki disease, ICD-10, Diagnostic codes

## Abstract

**Background:**

Pediatric rheumatic diseases are chronic illnesses that can cause considerable disease burden to children and their families. There is limited epidemiologic data on these diseases in East Africa. The aim of this study was to assess the spectrum of pediatric rheumatic diagnoses in an in-patient setting and determine the accuracy of ICD-10 codes in identifying these conditions.

**Methods:**

Medical records from Gertrude’s Children’s Hospital in Kenya were reviewed for patients diagnosed with “diseases of the musculoskeletal system and connective tissue” as per ICD-10 diagnostic codes assigned at discharge between January and December 2011. Cases were classified as “rheumatic” or “non-rheumatic”. Accuracy of the assigned ICD-10 code was ascertained. Death records were reviewed. Longitudinal follow-up of “rheumatic” cases was done by chart review up to March 2014.

**Results:**

Twenty six patients were classified as having a “rheumatic” condition accounting for 0.32% of patients admitted. Of these, 11 (42.3%) had an acute inflammatory arthropathy, 6 (23.1%) had septic arthritis, 4 (15.4%) had Kawasaki disease, 2 (7.7%) had pyomyositis, and there was one case each of septic bursitis, rheumatic fever, and a non-specific soft tissue disorder. No cases of juvenile idiopathic arthritis (JIA) were identified. One case of systemic lupus erythematosus was documented by death records. The agreement between the treating physician’s discharge diagnosis and medical records ICD-10 code assignment was good (Kappa: 0.769). On follow-up, one child had recurrent knee swelling that was suspicious for JIA.

**Conclusions:**

Pediatric rheumatic conditions represented 0.32% of admissions at a pediatric hospital in Kenya. Acute inflammatory arthropathies, septic arthritis and Kawasaki disease were the most frequent in-patient rheumatic diagnoses. Chronic pediatric rheumatic diseases were rare amongst this in-patient population. Despite limitations associated with the use of administrative diagnostic codes, they can be a first step in evaluating the spectrum of pediatric rheumatic conditions in Kenya and other countries in East Africa.

## Background

Pediatric rheumatic diseases are chronic illnesses that can cause considerable disease burden to children and their families. They are associated with the potential for physical disability, diminished quality of life and significant direct and indirect costs [1–3]. This underscores the importance of early diagnosis and treatment.

One of the aims of the “Bone and Joint Decade” (2000–2010) was to increase the recognition and understanding of the impact of musculoskeletal conditions throughout the world and to improve health-related quality of life for patients living with these conditions [4, 5]. Understanding the burden of pediatric rheumatic diseases is a critical first step in developing and implementing policies to improve patient outcomes. Multiple studies suggest that pediatric rheumatic conditions are not rare in Africa [6–11] yet there are limited data available from epidemiological studies. Although children represent 53% of the Kenyan population (38.5 million in 2010) [12], the spectrum and frequency of pediatric rheumatic disorders remains to be defined.

We undertook this study to assess the spectrum of pediatric rheumatic diagnoses among children hospitalized in 2011 at Gertrude’s Children’s Hospital (GCH) in Kenya and to determine the accuracy of the use of musculoskeletal ICD-10 diagnostic codes in identifying these conditions.

## Methods

### Setting

GCH is a private, not-for-profit hospital located in Nairobi, the capital of Kenya. The hospital serves the population of Nairobi and its environs with a combined population of approximately 4 million people [12]. It is a tertiary level hospital with a bed capacity of 103, an emergency department and 24 subspecialty clinics ranging from cardiology, neurology, pulmonology, dermatology, orthopedics etc. At the time this study was conducted, there were no pediatric rheumatologists in Kenya. GCH was selected because it is one of the largest pediatric centers in East Africa, it is the only dedicated pediatric hospital in Nairobi and it has an electronic medical record system that was implemented at the end of 2010.

At GCH, the primary physician makes a final diagnosis at discharge. Trained medical records’ clerks assign an International Classification of Disease 10^th^ edition (ICD-10) code based on the physician’s discharge diagnosis.

### Study procedures

Following ethical approval from the Institutional Review Boards of McGill University (Montreal, Canada) and Gertrude’s Children’s Hospital (Nairobi, Kenya), a retrospective cross-sectional study was undertaken. Medical records of patients diagnosed with “diseases of the musculoskeletal system and connective tissue” as per ICD-10 diagnostic codes (M00 to M99, “M-codes”) at discharge between January 1^st^ and December 31^st^ 2011 were identified by the electronic medical records system and selected for review. For in-patients, only the discharge diagnosis is recorded electronically. Data from the patient’s history, clinical examination, laboratory and microbiology investigations were extracted (AM) from paper charts using a structured case report form. After assessing the clinical information of each case, two rheumatologists (RS-pediatric rheumatologist and IC) classified cases as having a “rheumatic” condition if they had a chronic rheumatic disease; or if they had an acute inflammatory or infectious condition involving the joints, bursae, muscles, or ligaments. All other cases were considered “non-rheumatic”. Any discrepancies between the two rheumatologists were resolved by consensus.

For the “rheumatic” cases, we assessed the agreement between the ICD-10 code at discharge assigned by the medical records’ clerks with the discharge diagnosis given to the patient by the treating physician. In addition, we report the agreement between the diagnosis assigned by the treating physician at discharge and that considered by the rheumatologists upon chart review.

In addition, the medical records of all in-patients who died at GCH between January and December 2011were reviewed to ascertain if there were any fatal “musculoskeletal system and connective tissue” cases, which may not have been identified by the electronic medical record system. As well, to assess whether any of the patients diagnosed with a “rheumatic” condition in 2011 progressed to develop a chronic inflammatory disease, longitudinal follow-up of “rheumatic” cases was done by chart review up to March 2014 by a pediatric rheumatologist (RS).

### Statistics

Descriptive statistics were used. Kappa coefficients were reported to quantify the degree of agreement between the diagnoses given by the GCH treating physician, medical records’ clerks and rheumatologists.

## Results

**Fig. 1:**
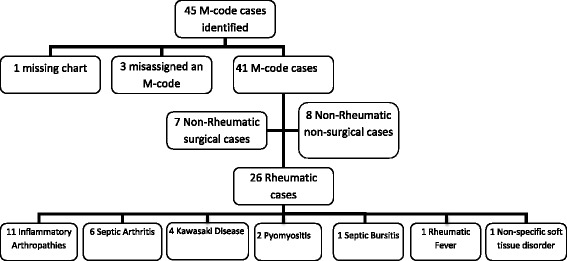
Spectrum of Pediatric Rheumatic Conditions

In 2011, there were a total of 8011 admissions at GCH of which 45 were assigned an “M-code” at discharge (Fig. 1). The medical records of 44 cases were reviewed (1 chart could not be retrieved). From the information documented in the medical records, 3 were misassigned as an “M-code”, 15 were classified as “non-rheumatic” and 26 were classified as having a “rheumatic” condition. The chart that could not be retrieved had a discharge diagnosis of scoliosis and would have likely been considered “non-rheumatic”.

Of the charts reviewed, surgical cases accounted for 7 of the “non-rheumatic” conditions. Examples of these included ganglion and dermoid cyst excisions. From the non-surgical “non-rheumatic” conditions, examples included musculoskeletal pain from sickle cell crises, neck pain from suspected meningitis, and torticollis from a viral infection. Only one patient was identified as being HIV positive; the patient with neck pain from suspected meningitis.

“Rheumatic” conditions accounted for 0.32% of the in-patient admissions at GCH in 2011. The median age of the “rheumatic” patients was 3 years (range: 3 months – 15 years); 58% (15/26) were male. “Rheumatic” diagnoses included: acute inflammatory arthropathies (*n* = 11, 42.3%), septic arthritis (*n* = 6, 23.1%), Kawasaki disease (KD) (*n* = 4, 15.4%), pyomyositis (*n* = 2, 7.7%), septic bursitis (*n* = 1, 3.8%), rheumatic fever (*n* = 1, 3.8%) and non-specific soft tissue disorder (*n* = 1, 3.8%). In the patients with an inflammatory arthropathy, HIV or TB arthritis was not suspected by the treating physicians.

The agreement between the ICD-10 code assigned by the medical records’ clerk and that of the treating physician for “rheumatic” conditions was 76.9% (20/26; Kappa: 0.769). The agreement between the ICD-10 code assigned by the treating physician and that given by the rheumatologist upon the medical record’s review for the “rheumatic” conditions was 84.6% (22/26; Kappa: 0.846). Perfect agreement was found between the clerk, treating physician and rheumatologist in cases of KD, rheumatic fever and pyomyositis (Table 1).

**Table 1:** Agreement between Rheumatic Diagnoses assigned by the Rheumatologist, Treating Physician and Records Clerk

Diagnosis by Rheumatologist	Agreement between Records Clerk and Treating Physician	Agreement between Treating Physician and Rheumatologist
	*n* (%) = 20/26 (76.9%)	*n* (%) = 22/26 (84.6%)
Inflammatory Arthropathies (11)	8 (72.73)	9 (81.82)
Septic Arthritis (6)	4 (66.67)	5 (83.33)
Kawasaki Disease (4)	4 (100)	4 (100)
Pyomyositis (2)	2 (100)	2 (100)
Septic Bursitis (1)	0 (0)	0 (0)
Rheumatic Fever (1)	1 (100)	1 (100)
Non-Specific Soft Tissue Disorder (1)	1 (100)	1 (100)

The charts of the 26 patients deemed to have a “rheumatic” condition were reviewed again in March 2014 to assess if any had progressed to develop a chronic inflammatory disease on follow-up. Only one patient had a recurrent episode of knee swelling suspicious for the diagnosis of juvenile idiopathic arthritis (JIA). No other patients had a chronic disease course. The discharge diagnosis of the child with suspected JIA was “reactive arthritis versus septic arthritis”. Upon initial chart review, and before the longitudinal follow-up, the patient had been categorized as an “acute inflammatory arthropathy” for this study.

From the 46 in-patient deaths from 2011, 45 charts were available and each was reviewed in detail. From this review, only 1 patient with a “rheumatic” condition was identified. This patient had systemic lupus erythematosus (SLE) and had died from sepsis. This patient had two admissions in 2011 and the discharge diagnosis assigned by the treating physician was “SLE”. However the medical records’ clerk had not assigned an “M-code” for either admissions and thus the electronic medical record system had not identified these admissions in the 2011 search.

## Discussion

Rheumatic conditions accounted for 0.32% of all admissions at GCH in 2011. Acute inflammatory arthropathies were the most common in-patient “rheumatic” diagnoses accounting for 42.3% of all “rheumatic” cases. These patients had a short duration of symptoms, suggesting a post-infectious etiology. No cases of JIA requiring admission were identified during the study period. Septic arthropathies and pyomyositis combined were as common as the inflammatory arthropathies. KD was the only vasculitis diagnosed and accounted for 15.4% of the “rheumatic” cases. One case of SLE was identified, but only when the death records were reviewed. This suggests the possibility that other patients with “rheumatic” conditions may have been missed due to administrative errors in diagnostic code assignment.

The follow-up chart review, performed in 2014 to assess the evolution of the 26 “rheumatic” cases identified in 2011, confirmed that only one patient had a chronic disease course with recurrent episodes of knee swelling suspicious for the diagnosis of JIA. However, this does not exclude the possibility that some of these patients were followed elsewhere. This is a limitation of a retrospective study.

The male predominance of the “rheumatic” cases (58%) reflects the disease entities seen in this study. Chronic rheumatic diseases such as JIA and SLE are more common among females however, these diseases were not found in this in-patient population and therefore a female predominance should not be expected. All 3 cases of pyomyositis and septic bursitis were male. These entities are more commonly seen post-trauma which would be expected to be more frequent in males. As well, KD is slightly more common in males and in this study, 3 of the 4 patients with KD were male.

In our study, there were no children admitted for JIA and only one for SLE. This could reflect the low prevalence of these conditions as a cause for hospital admission, the lack of awareness / expertise of pediatricians in diagnosing these conditions, the limited use of specific M codes, the underestimation of the real frequency of these diagnoses based on their ascertainment through ICD-10 codes (i.e. coding errors) or the limitation of M-codes in identifying multisystem diseases such as SLE. Furthermore, an in-patient population may not be the ideal target population to assess conditions like JIA, which may be more likely assessed in out-patient clinics.

In this study, we chose to use M-codes to identify cases. However it is possible, that other cases could have been captured by the use of other specific codes (i.e. renal or hematologic impairments for SLE) and that M-codes underestimate the frequency of multisystem diagnoses. In the case of SLE, despite having a specific diagnostic M-code, discharge diagnosis may reflect the patient’s reason for admission (i.e. renal failure) rather than the patient’s diagnosis, which is another limitation of the use of administrative diagnostic codes to identify cases. As well, it is important to note that misdiagnoses are difficult to capture no matter what tool is used to identify cases. For example, if the treating physician could not identify that the child being treated for fever, rash and pericarditis was a child with systemic JIA and the discharge diagnosis was pericarditis or viral illness, this case would not have been captured. The absence of these diseases may reflect the limitations associated with the use of administrative diagnostic codes to capture cases in addition to the lack of expertise of pediatricians to diagnose specific rheumatic diseases rather than the true absence of disease.

JIA and pediatric SLE cases have been identified in various countries across the African continent such as in Nigeria, South Africa and Zambia [6–11]. In the Nigerian studies, they identified 11 children with SLE who were admitted to a hospital over a 5 year period [9, 10]. The rates reported for SLE in our study appear lower than those reported in Nigeria. With regard to JIA, all previous reported cases reflected out-patient data and thus are not comparable.

The first pediatric case of KD was described in Africa in 1979 [13]. In our study, four cases of KD were identified over a one year period, which is comparable, or a bit higher, than a few of the larger African case series in the literature [14–16]. Rheumatic fever is still the most common cause of acquired cardiac disease in children in developing countries [17] yet in this study, only one case of rheumatic fever was admitted. It is possible that most cases were managed as out-patients.

In Kenya, there is both a private and public medical system with several private hospitals and one main public national referral hospital that provide pediatric care in Nairobi. In 2008, the Government of Kenya provided resources for 48% of the country’s health care facilities, while 49% was provided by the private sector (both for-profit and not-for-profit) [18]. Although private, GCH is the only dedicated pediatric hospital providing care exclusively to children; whereas the other hospitals have pediatric units. GCH has fewer but comparable in-patient admissions as compared to the main national public referral hospital (GCH: 8011 in 2011; public hospital: 9656 in 2007) [19]. We chose to undertake this study at GCH because it had the infrastructure to support this study particularly with the electronic medical records system. However, given that GCH only sees pediatric patients, it does not have access to adult rheumatology services. It is possible that if this study was conducted at the main public national referral hospital that has both adult and pediatric units, with access to adult rheumatology, more defined “rheumatic” cases could have been identified. In addition, given that GCH is a private hospital, the population that accesses it is different socioeconomically than those who can only access the public system.

It is important to highlight that at the time this study was conducted, there were no pediatric rheumatologists in Kenya. Global variability in the prevalence rates of pediatric musculoskeletal diseases has been attributed to varying levels of awareness of rheumatic diseases, combined with the lack of personnel and infrastructure to diagnose and manage these conditions [20]. This is particularly relevant in a setting where diverse and frequent entities such as infections, rickets and hemoglobinopathies [21] can lead to misdiagnosis and delays in the treatment of more rare rheumatic disorders. Olowu suggested that the paucity of pediatric rheumatologists, low level of lupus awareness, malaria endemicity (since malaria shares certain clinical features with lupus) contributed to misdiagnosis of lupus in the Nigerian pediatric population [10]. In his series, all SLE cases were misdiagnosed prior to referral (36.4% as malaria) suggesting a low level of awareness [10]. Prospective studies assessing rheumatic diagnoses made by pediatric rheumatologists now working in Kenya will be relevant to estimate the real burden of rheumatic conditions in this setting. We suspect that disease awareness and the presence of pediatric rheumatologists locally will improve case ascertainment and provide more accurate estimates of the frequency and spectrum of disease.

The agreement in the use of ICD-10 diagnostic codes by the records clerks and the diagnosis of the treating physician was good (Kappa 0.769). However there are challenges and limitations in using administrative databases in estimating disease prevalence. As expected, the more specific the treating physician’s discharge diagnosis, the more accurate was the assigned code as reflected by the 100% agreement for the diagnosis of KD. In contrast, the missed SLE coding likely occurred due to the use of an acronym (“SLE”) that was not interpreted by the medical record’s clerk. This highlights the importance of promoting a clear and precise record of discharge diagnoses by physicians as a way to increase the accuracy of administrative data in estimating frequencies of diagnostic categories.

The aim of pediatric global health priorities has been to reduce infant and childhood mortality. Infectious diseases and injuries account for 75% of cause-specific mortality among children 5–14 years of age [22]. Musculoskeletal disease only comprises 0.1% of all-cause mortality in the same age group [22]. Therefore musculoskeletal disease has not been considered a health priority for most African countries. Most medical efforts have been directed towards preventable diseases such as acute respiratory infections, malaria, measles, diarrhea, HIV, malnutrition and trauma [22]. However, as preventable diseases are better controlled and treated, there is an expected health priority shift towards chronic conditions including rheumatic diseases [20, 22]. Therefore the importance of rheumatic disease awareness and building medical expertise capacity cannot be overstated.

## Conclusions

To our knowledge, this is the first report of the spectrum of pediatric rheumatic conditions amongst in-patients in Kenya. Pediatric rheumatic conditions represented 0.32% of admissions at a pediatric hospital in Nairobi. Acute inflammatory arthropathies, septic arthritis and Kawasaki disease were the most frequent in-patient rheumatic diagnoses. Chronic pediatric rheumatic diseases were rare amongst this in-patient population. This may reflect the limitations associated with the use of administrative diagnostic codes and lack of expertise of pediatricians to diagnose specific rheumatic diseases rather than the true absence of disease. Increasing awareness of pediatric rheumatic diseases amongst health care professionals as well as the recruitment of pediatric rheumatologists to the region will allow for better identification of cases and estimation of the burden of these diseases among Kenyan children. Despite the limitations associated with the use of administrative diagnostic codes, they can be a first step in evaluating the spectrum and frequency of pediatric rheumatic conditions in Kenya and other countries in East Africa.

## Abbreviations

GCH: Gertrude’s Children’s Hospital
HIV: Human immunodeficiency virus
ICD-10: International classification of disease 10^th^ edition
JIA: Juvenile idiopathic arthritis
KD: Kawasaki disease
M-codes: Diagnostic codes for “diseases of the musculoskeletal system and connective tissue” as per ICD-10 (M00 to M99)
SLE: Systemic lupus erythematosus
